# Performance experimental data of a polymer electrolyte fuel cell considering the variation of the relative humidity of reactants gases

**DOI:** 10.1016/j.dib.2019.104727

**Published:** 2019-10-28

**Authors:** Mayken Espinoza-Andaluz, Jordy Santana, Tingshuai Li, Martin Andersson

**Affiliations:** a ESPOL Polytechnic, Escuela Superior Politécnica del Litoral, ESPOL, Facultad de Ingeniería Mecánica y Ciencias de la Producción, Centro de Energías Renovables y Alternativas, Campus Gustavo Galindo, Km. 30.5 Vía Perimetral, P.O. Box 09-01-5863, Guayaquil, Ecuador; b Escuela Superior Politécnica del Litoral, ESPOL, Facultad de Ingeniería Mecánica y Ciencias de la Producción, Campus Gustavo Galindo, Km. 30.5 Vía Perimetral, P.O. Box 09-01-5863, Guayaquil, Ecuador; c School of Materials and Energy, University of Electronic Science and Technology of China, 2006 Xiyuan Ave, West Hi-Tech Zone, Chengdu, Sichuan, China; d Department of Energy Sciences, Faculty of Engineering, Lund University, P.O. Box 118, Lund, Sweden

**Keywords:** Fuel cell, Relative humidity, Performance, Voltage, Power density

## Abstract

The data collected in this article is based on a performance test of a polymer electrolyte fuel cell (PEFC). The behavior the different parameters of a PEFC is analyzed considering different aspects relative to the inlet gases temperatures. The fuel cell was evaluated by means of a current sweep at different percentages of relative humidity between the feed gas and the cell. The relative humidity values were established by means of the temperature setting. The data presented show the experimental response of the cell in real time, which can be used to perform a depth analysis or they can be a starting point for material and performance investigation. In addition, charts presenting the voltage and power density behavior as a function of the volumetric flows of the anode (H2) as well as cathode (O2). The data presented in this article are originally from our research performed in [1].

**Table:** Specifications Table

Subject	Energy
Specific subject area	Polymer Electrolyte Fuel Cell
Type of data	Tables Charts
How data were acquired	Current Sweep – Experimental data taken from fuel cell test system
Data format	Raw and analyzed
Parameters for data collection	The gases used were H_2_ and O_2_, the inlet pressure are kept at 55 psig. The water employed was ASTM Type I, and the membrane electrode assembly (MEA) was tested at 80 °C. Data were collected by varying the percentages of relative humidity in function of the inlet temperature of gases reactants.
Description of data collection	Data were collected through the Fuel Cell® software, which is directly connected to the control system tester of the fuel cell.
Data source location	Laboratory of Renewable Energy Sources, Escuela Superior Politécnica del Litoral, Ecuador. (Lab-FREE)
Data accessibility	The raw data files are provided in the Data in brief Dataverse, https://doi.org/10.7910/DVN/N1RU9X [2].
Related research article	Espinoza-Andaluz M et al., Empirical correlations for the performance of a PEFC considering relative humidity of fuel and oxidant gases, International Journal of Hydrogen Energy, https://doi.org/10.1016/j.ijhydene.2019.09.098. In press [1].

**Table:** 

**Value of the Data** • The experimentally obtained data shows the performance of a PEFC at different relative humidity values. The PEFC behavior is required to design improvements from a cell scale point of view. • The graphic representation of the data helps to observe the behavior of a PEFC as a function of relative humidity enabling interpretation and ability to carry out new investigations based on the graphics shown in this document. • Experimental data collection takes considerable time, i.e., these data give a focus on the cell behavior without having to perform the test themselves.

## 1 Data description

The shared data are obtained from an experimental test where a PEFC was evaluated at different relative humidity conditions by means of a current sweep. This was achieved by configuring the temperatures of the feed gases and the cell in different proportions. Due to the great amount information. The data are sharing online on the data repository [2], while some relevant diagrams for their analysis are showed in this article. The volumetric flow in the anode and cathode side were considered as independent variables while the voltage and power density were taken as the dependent variable.

### 1.1 Voltage as power density as a function of the volumetric flow

**Fig. 1:**
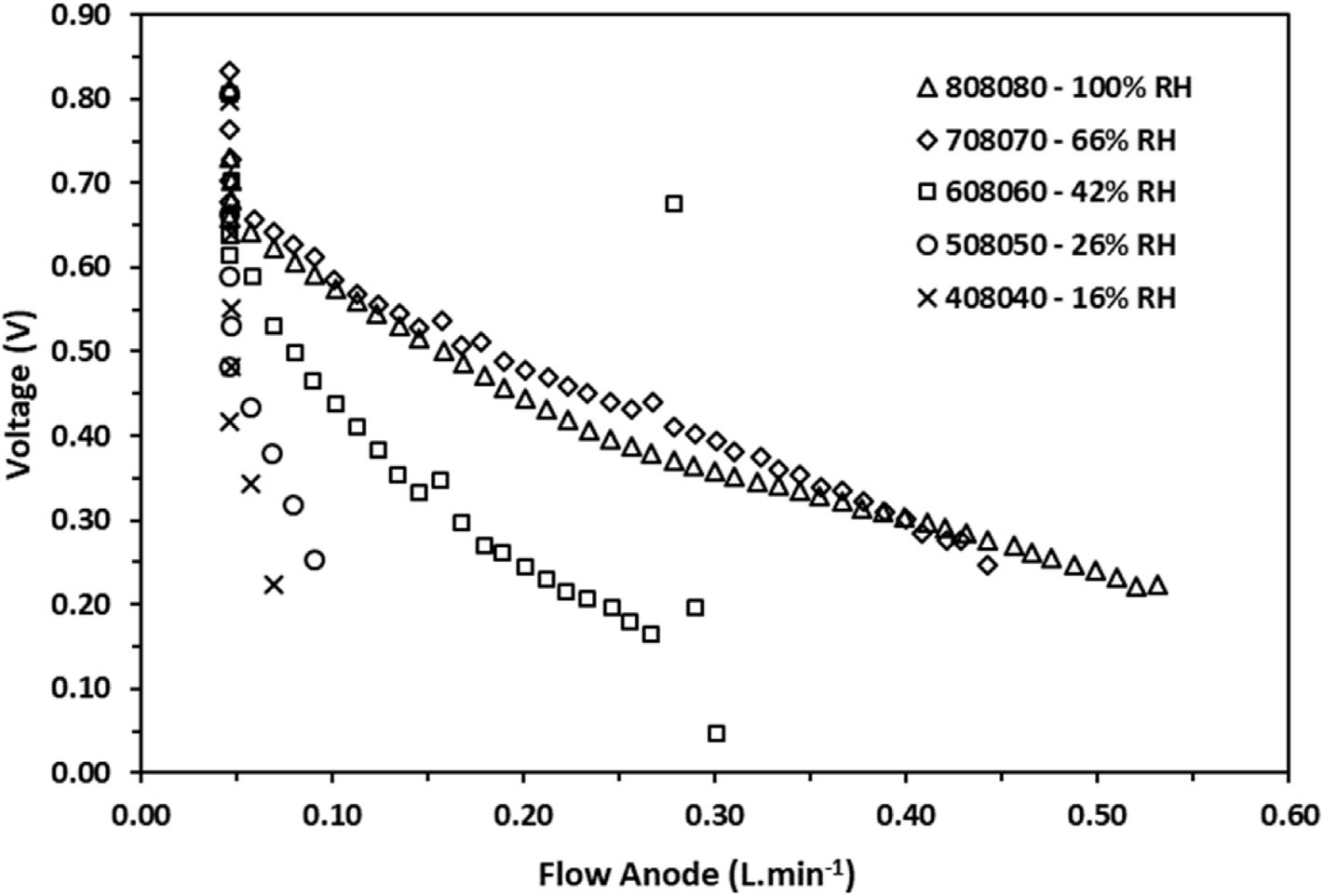
Voltage of a single cell as function of the gas flow at the anode side measured at several percentages of relative humidity.

**Fig. 2:**
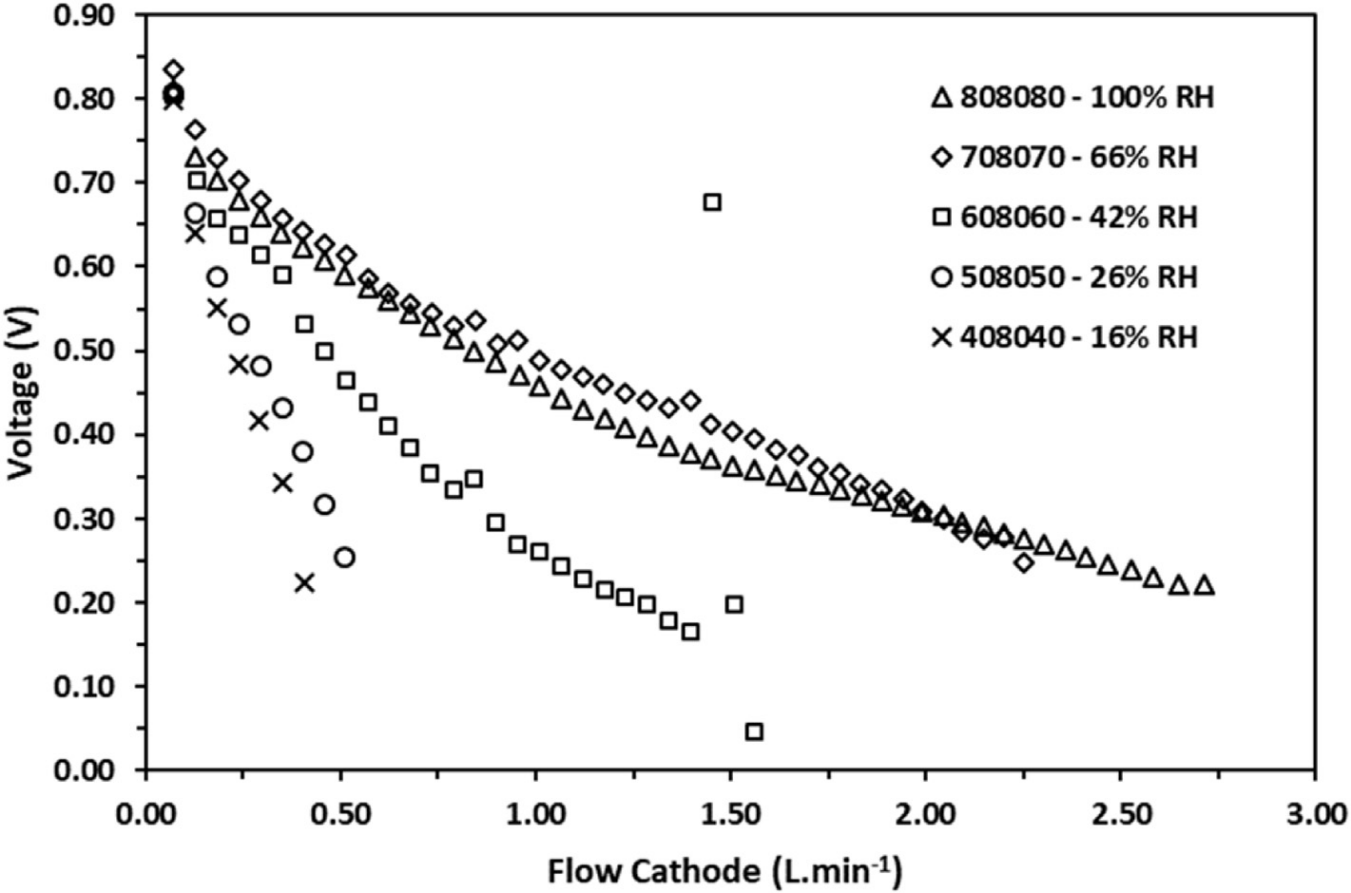
Voltage of a single cell as function of the gas flow at the cathode side measured at several percentages of relative humidity.

**Fig. 3:**
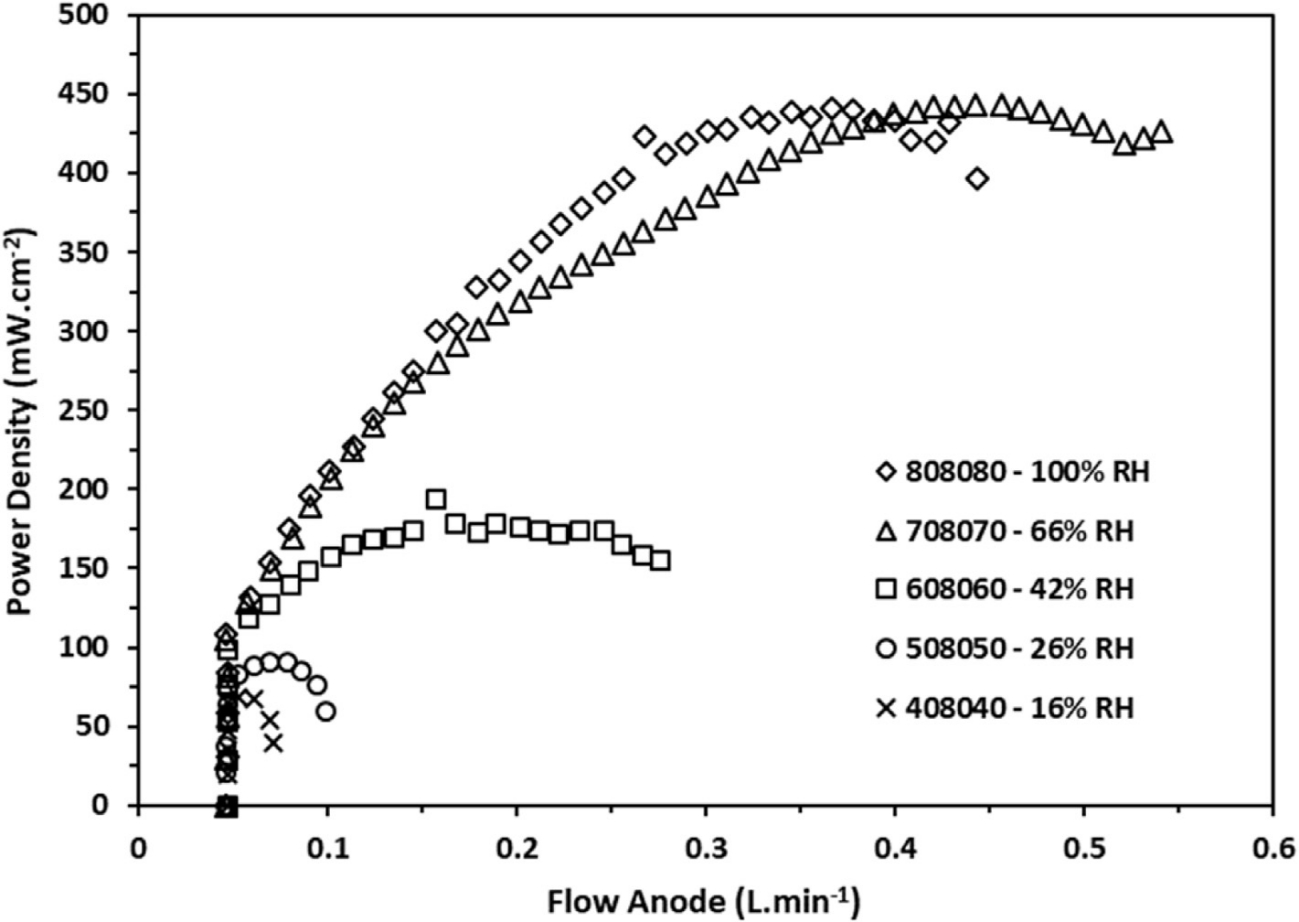
Power density of a single cell as function of the gas flow at the anode side measured at several percentages of relative humidity.

**Fig. 4:**
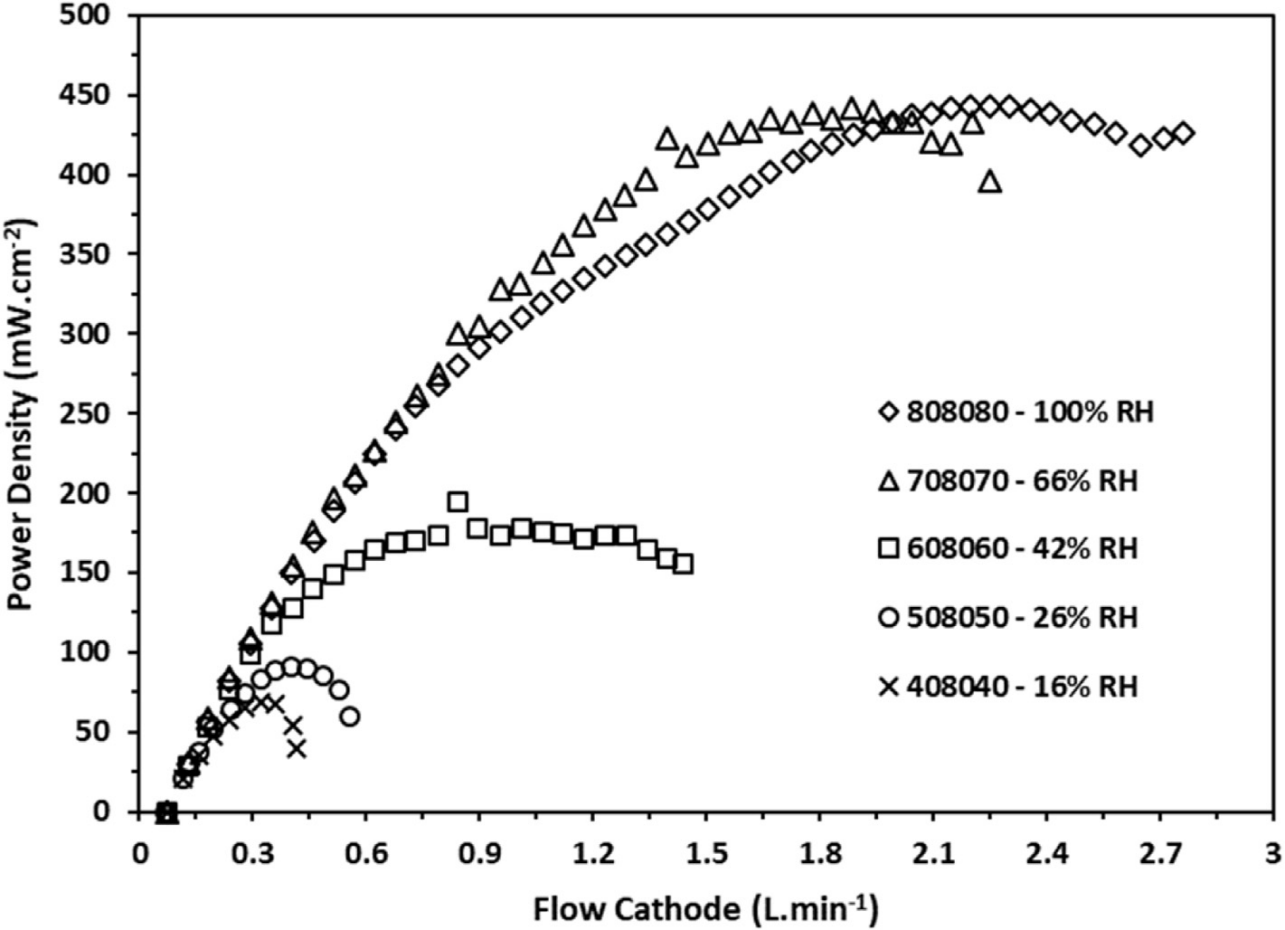
Power density of a single cell as function of the gas flow at the cathode side measured at several percentages of relative humidity.

In Fig. 1, Fig. 2 it can be observed that the behavior of the voltage as a function of the volumetric flow for various relative humidity conditions. The experimental data were obtained by configuring the volumetric flow values using the computational tool for setting the conditions. The stoichiometric ratios employed for the data collection were established at 1.2× for the H_2_ flow and 2.5× for the O_2_, both flows were configured based on the applied load. Similarly, in Fig. 3, Fig. 4 the behavior of the power density is shown as a function of the volumetric flow of the anode and the cathode flow fields.

Subsequently in Table 1, a brief part of experimental data collected in the fuel cell performance test is shown, specifically the data shown are for a relative humidity of 16%, having as the Anode/Cell/Cathode temperature settings with values of 40/80/40 respectively. The remaining data for the following relative humidity and temperature settings are displayed in the data repository [2]. The data shown were recorded according to the time step, where the parameters as current, current density, power density, cell voltage, anode temperature (hydrogen inlet), cell temperature, cathode temperature (oxygen inlet), volumetric flow in the anode and cathode, were collected directly by using a fuel cell data acquisition.

**Table 1:** Experimental data collected in the fuel cell performance test at 16% of relative humidity, i.e., Anode/Cell/Cathode temperatures are 40/80/40 respectively.

Time (s)	Current (A)	Current Density (mA.cm^−2^)	Power Density (mW.cm^−2^)	Voltage (V)	Temp. Anode (°C)	Temp. Cell (°C)	Temp. Cathode (°C)	Flow Anode (l.min^−1^)	Flow Cathode (l.min^−1^)
60	0	0.000	0.0	0.797	40	80	40	0.0470	0.0716
120	0.253	10.115	7.5483	0.746	40	80	40	0.0477	0.0870
180	0.501	20.031	14.1320	0.706	40	80	40	0.0473	0.1017
240	0.751	30.035	20.1330	0.670	40	80	40	0.0472	0.1145
300	1.000	39.988	25.5790	0.640	40	80	40	0.0476	0.1288
360	1.249	49.956	30.6690	0.614	40	80	40	0.0468	0.1422
420	1.496	59.833	35.3510	0.591	40	80	40	0.0469	0.1564
480	1.751	70.046	39.8770	0.569	40	80	40	0.0471	0.1706
540	2.002	80.071	44.0980	0.551	40	80	40	0.0477	0.1828
600	2.250	89.985	47.7790	0.531	40	80	40	0.0468	0.1973
660	2.503	100.130	51.5400	0.515	40	80	40	0.0471	0.2129
720	2.749	109.960	54.9260	0.499	40	80	40	0.0469	0.2252
780	2.997	119.890	57.9080	0.483	40	80	40	0.0473	0.2381
840	3.249	129.950	60.8590	0.468	40	80	40	0.0473	0.2510
900	3.498	139.930	63.2700	0.452	40	80	40	0.0467	0.2655
960	3.745	149.800	65.1920	0.435	40	80	40	0.0469	0.2800
1020	3.999	159.950	66.7140	0.417	40	80	40	0.0470	0.2924
1080	4.251	170.040	67.8990	0.399	40	80	40	0.0500	0.3046
1140	4.497	179.860	68.4970	0.381	40	80	40	0.0528	0.3203
1200	4.750	189.980	69.1510	0.364	40	80	40	0.0557	0.3344
1260	5.000	200.010	68.6420	0.343	40	80	40	0.0581	0.3497
1320	5.246	209.860	67.7860	0.323	40	80	40	0.0611	0.3610
1380	5.493	219.720	63.9450	0.291	40	80	40	0.0636	0.3752
1440	5.749	229.950	60.4080	0.263	40	80	40	0.0671	0.3904
1500	5.998	239.920	53.6770	0.224	40	80	40	0.0698	0.4060
1560	6.251	250.030	39.4610	0.158	40	80	40	0.0710	0.4172

## 2 Experimental design, materials, and methods

### 2.1 Data acquisition

The data acquisition system consists of a Fuel Cell® software, a GPIB Instruments control device cable and a fuel cell test System from Scriber®. The use of the mentioned tools allows us to control the input variables of the experiment from a peripheral device. Variables such as the inlet temperatures of the H_2_/O_2_ feed gases were controlled, the respective volumetric flows and the current load applied to the cell were tested as every step in the process of the data collection. Also, by means of the computational tool it is possible to control the opening of the valves of the system, and to configure the different types of experiments that can be carried out with the equipment. For more information on the fuel test System readers are referred to Ref. [3].

### 2.2 Experimental design

Initially, an inlet pressure of the H_2_/O_2_ feed gases was set at 55 psig, N_2_ was used as a purge gas to keep the flow distribution system clean and avoid the reactions with the other reagents. The water used was ASTM type I (with 18 MΩ cm^−1^ minimum resistivity), because the membrane electrode assembly should be prevented from contamination. The evaluation of the performance of the cell in several conditions of relative humidity was carried out to perform an analysis based on the maximum efficiency temperature of the cell, i.e., 80 °C [4]. The mentioned temperature is kept constant, then a configuration was made for the gases entering to the systems. Temperature of the gases are established in the range of 40 °C–80 °C, in steps of 10 °C. This temperature step corresponds to the double of the considered in a previous research that involve a PEFC with similar characteristics [5]. The relative humidity calculation was obtained as the ratio between the saturation pressure of the cell at 80 °C and the saturation pressure of the feed gases at their corresponding inlet temperature. This analysis can be carried out since the system has humidifier tanks which saturate the feed gases to their dew point, according to the set temperature. This experiment was designed considering some specifications described in Ref. [6]. The temperature of the gases play an important role during the energy conversion process specially when phase change occurs [7].
